# Effectively Communicating the Uncertainties Surrounding Ebola Virus Transmission

**DOI:** 10.1371/journal.ppat.1005097

**Published:** 2015-10-29

**Authors:** Andy Kilianski, Nicholas G. Evans

**Affiliations:** 1 BioDefense Branch, BioSciences Division, Edgewood Chemical Biological Center, Aberdeen Proving Ground, Maryland, United States of America; 2 Department of Medical Ethics and Health Policy, University of Pennsylvania, Philadelphia, Pennsylvania, United States of America; The Fox Chase Cancer Center, UNITED STATES

## Abstract

The current Ebola virus outbreak has highlighted the uncertainties surrounding many aspects of Ebola virus virology, including routes of transmission. The scientific community played a leading role during the outbreak—potentially, the largest of its kind—as many of the questions surrounding ebolaviruses have only been interrogated in the laboratory. Scientists provided an invaluable resource for clinicians, public health officials, policy makers, and the lay public in understanding the progress of Ebola virus disease and the continuing outbreak. Not all of the scientific communication, however, was accurate or effective. There were multiple instances of published articles during the height of the outbreak containing potentially misleading scientific language that spurred media overreaction and potentially jeopardized preparedness and policy decisions at critical points. Here, we use articles declaring the potential for airborne transmission of Ebola virus as a case study in the inaccurate reporting of basic science, and we provide recommendations for improving the communication about unknown aspects of disease during public health crises.

## Author Summary

Basic scientific research is now considered an integral component of the fight against emerging infectious diseases like Ebola virus. The recent Ebola outbreak, however, demonstrates how the ineffective communication of basic science can stoke public panic more than it provides helpful tools to responders; basic science trades in probabilities and uncertainty, while public communication tends to favor more categorical claims. Here, we discuss the ethics of communicating scientific results, using, as a case study, the recent controversy over whether basic life sciences research demonstrates that Ebola could become transmissible via airborne respiratory droplet nuclei—popularly known as a virus becoming “airborne.” We show how the science does not demonstrate this possibility, despite claims made in the popular and scientific press. We then recommend that uncertain scientific results in the context of public health crises ought to be communicated with humility, an emphasis on what is unknown, and a clear outline of the kinds of evidence that would give proof to controversial claims.

## Introduction

The current Ebola virus disease (EVD) outbreak due to Ebola virus (EBOV) infection continues to affect the West African nations of Liberia, Sierra Leone, and Guinea. This outbreak has highlighted the uncertainties surrounding many aspects of ebolavirus virology, including how the virus is transmitted. High mortality and historical case fatality rates, combined with graphic descriptions of the pathology of EVD caused by Ebola virus, have stoked public panic, and a lack of available clinical and public health expertise in treating EVD has led the media to turn to infectious disease scientists for information. This represents a significant involvement in public health efforts by the virology and infectious disease community, on par with the severe acute respiratory syndrome outbreak of 2002–2003 and the 2009 H1N1 influenza pandemic [1,2].

A majority of the information presented to news sources has been accurate and factual, helping to inform the public about EVD. However, a small subset of information discussed by scientists has fanned flames of panic among the public, government officials, and policy makers. As professionals with expertise and knowledge, scientists’ position of power over the general public (and media) generates a responsibility to convey accurate information [3]. Particularly in areas of scientific inquiry that can be reasonably expected to create a significant public panic—such as commentary on the spread of EVD—scientists must take extra care to convey the radical uncertainty that often surrounds knowledge of emerging infectious diseases.

## “Airborne” Ebola: Potential Pandemic or Tempest in a Teacup?

A particularly concerning lapse in accurate reporting lies in EBOV’s transmission. In September 2014, an op-ed piece published in the *New York Times* (*NYT*), titled “What We’re Afraid to Say About Ebola,” [4] asserted that the virology community writ large was “loath to discuss openly but are definitely considering in private: that an Ebola virus could mutate to become transmissible through the air.” Recent studies have examined the mutation rate of the outbreak and determined that the current strains do not have increased rates of mutation compared to previous outbreaks and that these mutations have no apparent effect on virulence [5–7]. The claims of the op–ed were repeated in February 2015: an article published in the journal *mBio* [8] provided a thorough review of the questions still remaining for the current EBOV outbreak. Toward the conclusion of the article, the authors state:

It is very likely that at least some degree of Ebola virus transmission currently occurs via infectious aerosols generated from the gastrointestinal tract, the respiratory tract, or medical procedures, although this has been difficult to definitively demonstrate or rule out, since those exposed to infectious aerosols also are most likely to be in close proximity to and in direct contact with an infected case [8].

Both articles were syndicated widely, with the *NYT* piece being covered internationally [9–11] and the *mBio* article receiving extensive coverage within the United States [12–14]. While a number of agencies questioned and critiqued the claims [15,16], large media outlets circulated the claims of the op-ed and paper with little to no accompanying criticism.

The *mBio* article, in particular, draws conclusions that eschew distinctions between aerosolized droplets and airborne droplet nuclei, to dramatic effect. Aerosol transmission involves any transmission mediated by aerosol droplets. Droplets are typically large in size (>5 μm) and cannot travel beyond the immediate vicinity of an infected individual, and they typically are only present in the later stages of EVD [17]. EBOV aerosol transmission is not a given, however, with studies in animal models providing conflicting evidence about the likelihood of transmission [18–20]. Even less can be said about the airborne transmission of EBOV between humans—that is, transmission that involves droplet nuclei (<5 μm) that can remain suspended in the air for prolonged durations. To date, there have been no studies conclusively demonstrating EBOV aerosol transmission. This is an obvious knowledge gap that needs to become an investigative priority for future response efforts.

Osterholm et al. note these distinctions in the body of their review, yet their conclusions do not match the nuances of aerosol transmission. Moreover, a key claim of theirs—that there is a series of unexplained transmission events in the epidemiological data collected on Ebola virus—does not entail conclusive evidence of novel transmission. There are a number of other plausible reasons for these unexplained events; the most obvious of these is that a self-report of contact history can be unreliable. We don’t discount the importance of these points in the context of a scientific review; rather, the reasons for questioning EBOV’s transmission mechanisms do not entail the claim that airborne transmission of aerosolized respiratory droplets is likely (or worth considering yet as a matter of public policy).

While the science supporting or refuting the aerosol transmission of EBOV remains unclear, the word “aerosol” carries significant weight in public discourse. It is far from clear whether media coverage of these articles has articulated the speculative nature of the claims made by Osterholm et al. Moreover, attempts to push back against these claims after the fact are likely to be met with suspicion; public trust in science is at an all-time low [21,22]. Given what “aerosol” means to members of the public without an infectious disease background, merely suggesting aerosol spread as a route of EBOV transmission without further definition is misleading.

To date, evaluations of the effectiveness of scientific communication during the current EBOV outbreak have been focused on the larger organizations (e.g., WHO, the Centers for Disease Control and Prevention [CDC], etc.) and their shortcomings [23]. However, the basic science community has had a large input into the public discussion surrounding EBOV. The lay public, policy makers, and officials all turn to basic scientists at times of uncertainty, and that creates a difficult situation that should be managed with caution.

## Managing Uncertainty

Matters of uncertainty are particularly fraught for scientific communication. Low probability events—or even logically or biologically possible events that cannot have probabilities assigned to them, such as a change of EBOV’s transmission characteristics—are likely to cause confusion and panic. There is a well-established literature on the psychology of human decision-making, suggesting that humans assign higher subjective probabilities to events of great significance [24]. In these circumstances, possibilities become probabilities, and probabilities become likelihoods.

The upshot of this is clear. Simply articulating that we should not assume that an event won’t happen becomes a tacit endorsement that the event could happen, along with all the psychological baggage that entails. The startling panic that has occurred in the context of the current Ebola virus disease outbreak is evidence of what does happen when the mere possibility of something occurring provokes a disproportionate response.

The responsibility to communicate well can be derived from a number of sources; for our purposes, the central concern is the vulnerability of others to misunderstandings brought about by an improper communication of risk. Scientists, particularly in infectious disease, generate claims and evidence that are probabilistic in nature. Those scientists, presumably, have gained the skills to interpret scientific evidence and understand what the information does—and doesn’t—tell us about the world. These skills, when applied to a broad public forum, generate a responsibility for scientists to do their part to prevent misinformation from spreading; unfortunately, they can do as much harm as good without responsible communication practices.

## Moving Forward

When communicating more broadly—and in a world of open-source publications, “broadly” should be taken as a given—different strategies are required to discharge the responsibility to communicate accurately. The first is epistemic humility: probabilistic claims should be couched in terms that emphasize the risks of false-positives and false negatives. This is particularly important when we are unable to determine the likelihood of our claims being right—or wrong. When there are no probabilities to assign, this lack of knowledge should be emphasized.

Second, language should be chosen to demonstrate relative, rather than absolute, credence in certain claims. In the context of the *mBio* paper, it would be more useful to make claims about the likelihood that unexplained transmissions were due to errors in reporting, or recall, rather than a new mode of transmission. It is true that we can’t demonstrate that these transmissions weren’t airborne, but it is likely that we can make a partial ordering of scenarios and identify airborne transmission from within a range of possibilities.

Fortunately, the current EBOV outbreak has not threatened the target audience of these opinions (e.g., United States, Canada, and Mexico), thus limiting the political will to engage in expensive or controversial (e.g., enforced quarantine) public health practices in the name of combating an airborne EBOV. Nonetheless, these commentaries have tremendous potential to impact public health policy and emergency planning moving forward. The potential of EBOV spread via aerosol routes drastically changes health care and public health interventions.

The impact of an airborne EBOV on public health and clinical medicine would entail large structural changes in contact tracing, the use of PPE, and patient isolation. It could also deter people from seeking medical treatment due to concerns about increased transmissibility, which would disrupt efforts to diagnose and treat infected individuals early. Our best response to an Ebola virus outbreak involves rapid contact tracing; a decrease in help-seeking behavior could fuel an outbreak if transmission chains are hidden from public health authorities. Given the immense costs and the possibility of public panic, it is imperative that more research is done before decisions are made. Until then, the life sciences must ensure that when public statements are made to the media, policy makers, and the general public, the most accurate depiction of scientific knowledge—including uncertainty—is conveyed. The potential impact of the Ebola virus transmission articles that we’ve discussed is illustrated by the public panic that surrounds the articles and the Ebola virus disease more generally. It is up to the scientific community as a whole to ensure that science drives discussions of emerging infectious disease and outbreak management in the future, and that it does so responsibly.
